# Antimicrobial coatings based on chitosan to prevent implant-associated infections: A systematic review

**DOI:** 10.1016/j.isci.2021.103480

**Published:** 2021-11-22

**Authors:** Rita Teixeira-Santos, Marta Lima, Luciana C. Gomes, Filipe J. Mergulhão

**Affiliations:** 1 LEPABE - Laboratory for Process Engineering, Environment, Biotechnology and Energy, Faculty of Engineering, University of Porto, Rua Dr. Roberto Frias, 4200-465 Porto, Portugal

**Keywords:** Microbiofilms, Materials science

## Abstract

Despite the advancements in material science and surgical techniques, the incidence of implant-associated infections (IAIs) has increased significantly. IAIs are mainly caused by microbial adhesion and biofilm formation on implant surfaces. In this study, we aimed to evaluate and critically discuss the antimicrobial efficacy of chitosan-based coatings to prevent the occurrence of IAIs. For this purpose, a PRISMA-oriented systematic review was conducted based on predefined criteria and forty studies were selected for qualitative analysis. Results indicated that chitosan (CS) association with enzymes and antimicrobial peptides improves its antimicrobial activity and extends its use in a broad range of physiological conditions. Likewise, CS association with polymers resulted in enhanced antimicrobial and anti-adhesive coatings with desirable properties, such as biocompatibility and durability, for implantable medical devices (IMDs). These findings can assist researchers in the design of new CS coatings for application in IMDs.

## Graphical abstract

**Figure:**
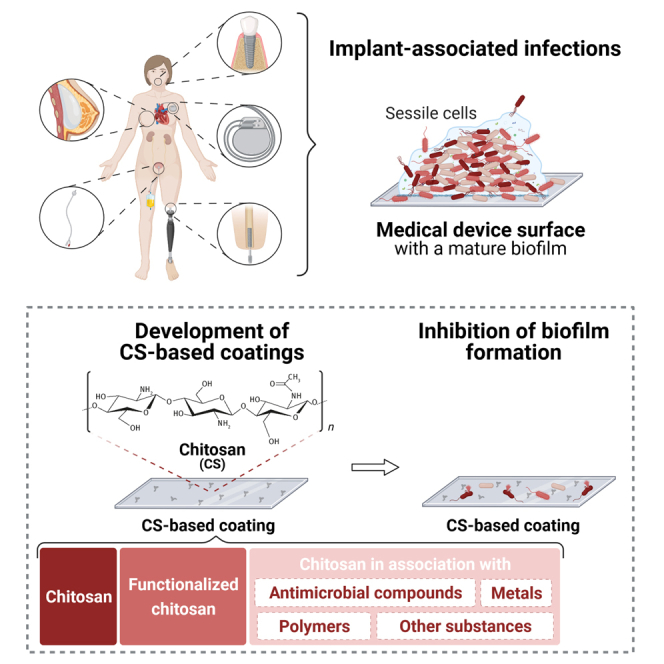

## Introduction

Of the 2 million healthcare-associated infections (HAIs) reported annually in the United States, about 60%–70% are attributed to some type of implanted medical device (Bryers, 2008; Vanepps and Younger, 2016). Given the uncertainty in diagnosis, implantable device-related infections may be responsible for more device-related complications and morbidity than previously thought (Vanepps and Younger, 2016), creating a massive economic and social burden. The annual costs directly associated with implant-associated infections (IAIs) are over $3 billion in the United States (Hetrick and Schoenfisch, 2006) and £7–11 million in the United Kingdom (Harris and Richards, 2006). In terms of attributable mortality, IAIs are highly device-dependent but can range from <5% for devices such as mammary implants and joint prosthesis to >25% for heart assist devices (Weinstein and Darouiche, 2001). Despite the groundbreaking developments in material sciences and process improvements at the time of implantation, these numbers are likely to grow owing to increased rates and types of device utilization (e.g., pacemakers, prosthetic joints, and catheters), the aging of the population, and the increasing frequency of comorbidities leading to immunocompromised states (Vanepps and Younger, 2016).

IAIs are mainly caused by biofilm development on implant surfaces (Kuehl et al., 2016). All medical devices are susceptible to microbial colonization and infection (Bryers, 2008), and the specific biofilm-forming organisms that cause device-related infections depend on the location of the device. The most common organism isolated from infected medical devices is coagulase-negative staphylococci, typically *Staphylococcus epidermidis*. Together with *Staphylococcus aureus*, they account for 50%–60% of the causative bacteria (Tande and Patel, 2014). Owing to their ability to rapidly form biofilm on the inert surface, they manage to grow and impair not only the defensive mechanisms of the host but also the bactericidal activity of antibiotics (Donlan and Costerton, 2002). Other important but less common pathogens include *Enterococcus* species, *Candida* spp., *Escherichia coli*, and *Klebsiella* spp., especially in intravascular devices, prosthetic joints, and urinary tract devices (Vanepps and Younger, 2016). Current treatment of IAIs includes the delivery of high-dose antimicrobials according to the severity of infection, and if symptoms persist revision surgery is needed. In many cases, conventional antimicrobial agents are ineffective at treating these infections owing to the presence of biofilms and the increasing prevalence of antimicrobial-resistant pathogens (Khatoon et al., 2018). Thus, the development of new strategies to prevent device colonization and biofilm formation is essential.

**Figure 1:**
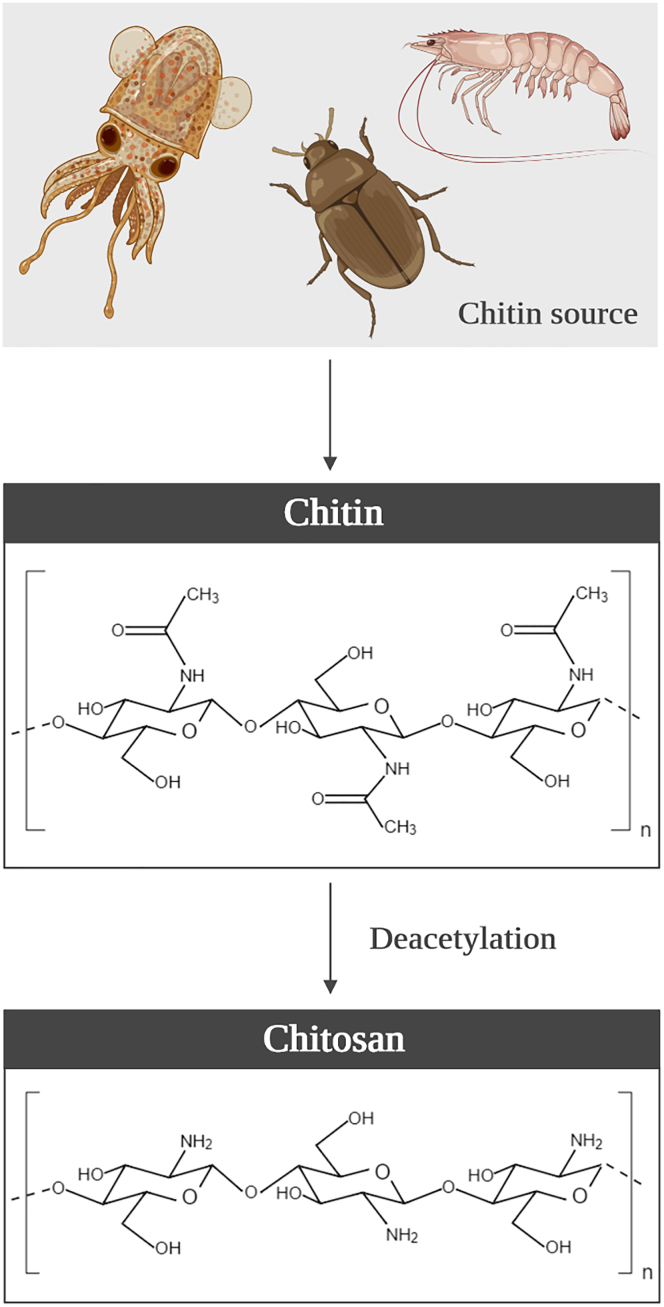
Chitosan origin Schematic representation of the chitin sources, and the chemical structure of chitin and its derivate chitosan.

A possible strategy to interfere with microbial adhesion is coating the implant with compounds that can generate anti-adhesive (e.g., polymers), contact-killing (e.g., antimicrobial peptides), or antimicrobial-releasing (e.g., metals and biocides) surfaces (Adlhart et al., 2018; Khatoon et al., 2018). From the compounds recently tested, chitosan (CS) and its derivatives stood out for their wide-spectrum antimicrobial activity and clear effectiveness against planktonic and biofilm cells (Khan et al., 2020). CS is a semi-natural polymer derived from chitin, the second most abundant natural amino polysaccharide on Earth after cellulose (Oyatogun et al., 2020) and, excluding proteins, the natural compound with the largest nitrogen content (Ravi Kumar, 2000). Chitin is usually obtained from the cell walls of fungi and exoskeletons of crustaceans and insects through demineralization and deproteinization processes. Although in its crude form chitin has poor solubility and low reactivity (Goy et al., 2009), its structure can be modified by removing the acetyl groups (bonded to amine radicals in the C2 position on the glucan ring, Figure 1) by chemical hydrolysis in alkaline solution at elevated temperatures to generate an improved deacetylated form (Goy et al., 2009; Zargar et al., 2015). When chitin is deacetylated by 40%–50%, the chitosan—a copolymer of N-acetyl-D-glucosamine and D-glucosamine units with β binding at positions 1 and 4—is produced (Figure 1) (Xing et al., 2016; Zargar et al., 2015). CS is soluble in dilute aqueous acidic solutions below its pKa (∼6.3), in which it can convert glucosamine units (-NH_2_) into the soluble protonated form (-NH_3_^+^). In addition, its reactive functional groups (amino group at the C2 position of deacetylated units and hydroxyl groups at the C6 and C3 positions) can experience chemical derivatization, allowing to modulate CS solubility, and thus improving its biocompatibility and antimicrobial properties (Goy et al., 2009).

Nowadays, the main commercial production of chitosan, 10^9^–10^10^ tons per year, is based on crustacean shells, which are an abundant and readily available byproduct of the seafood industries (Oyatogun et al., 2020). Thus, chitosan synthesis is an eco-friendly and economically viable process. CS market size was valued at over $1.2 billion in 2015 and was forecasted to reach $4.2 billion by 2021, at a compound annual growth rate of 15.4% (Oyatogun et al., 2020). This biopolymer has attracted considerable interest for biomedical applications owing to its outstanding biological properties, such as biocompatibility, biodegradability, non-toxicity, non-allergenic behavior, and antimicrobial activity (Anitha et al., 2014; D'almeida et al., 2017). It can be prepared in different forms, such as gels, nanoparticles, fibers, membranes or sponges, allowing a large variety of medical applications such as tissue engineering (Kim et al., 2008; Tan et al., 2009), wound healing (Patrulea et al., 2015), drug delivery (Li et al., 2018), or skin regeneration (Feng et al., 2021).

**Figure 2:**
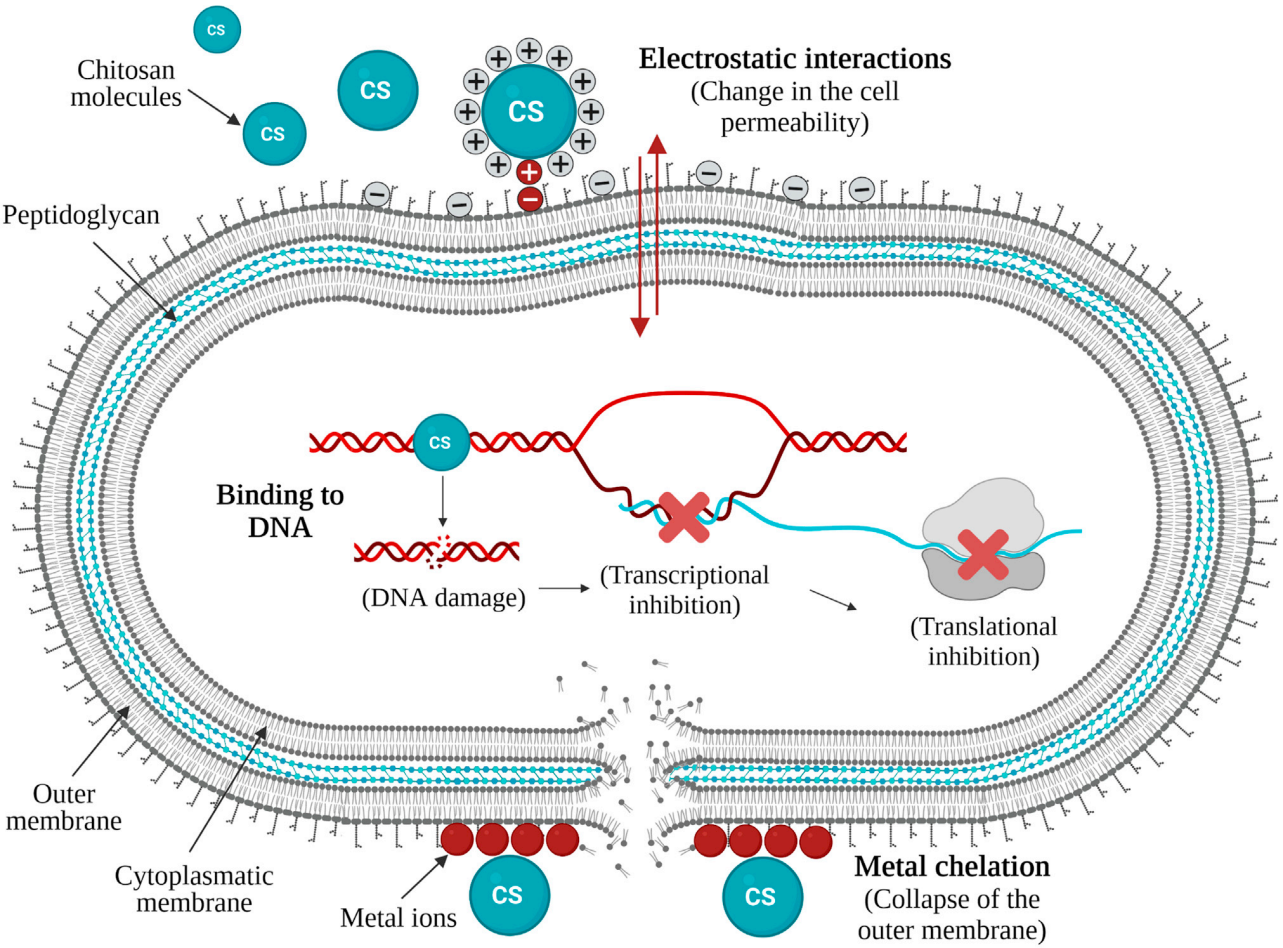
Antibacterial activity of chitosan Schematic representation of possible antibacterial mechanisms of chitosan and its derivatives: cell wall charge disruption, metal chelation, and cytoplasmic DNA complexation.

Chitosan and its derivatives are active against gram-negative and gram-positive bacteria, filamentous fungi, and yeasts, but show lower toxicity toward mammalian cells (Tikhonov et al., 2006), representing very attractive materials for the design of surface coatings of medical application. The mode of action of CS is not entirely known, but three main mechanisms are suggested for inhibition of bacterial growth (Figure 2): (i) cell wall charge disruption, (ii) metal chelation, and (iii) complexation with DNA. In the first, positively charged CS molecules react with the anionic phosphate groups of phospholipids found on the bacterial cell wall by using their NH_3_^+^ amino group, thereby leading to changes in the cell permeability and eventual release of the cellular content (Li et al., 2010, 2015). In the second mechanism, the amino groups in the CS molecules are responsible for the uptake of metal cations (e.g., Ca^2+^ or Mg^2+^) by chelation, destroying the integrity of the bacterial cell wall (Chung and Chen, 2008; Li et al., 2010). Another mechanism involves the interaction of diffused hydrolysis products with microbial DNA, which leads to the inhibition of the messenger RNA (mRNA) and protein synthesis (Hosseinnejad and Jafari, 2016). The antibacterial activity of chitosan may result from a combination of different mechanisms (Chung and Chen, 2008). Chitosan and its derivatives are dependent on many factors to display their antimicrobial properties, including environmental parameters such as pH, type of microorganism, and neighboring components, and its structural conditions such as molecular weight, degree of deacetylation, derivative form, concentration, and the source (Kong et al., 2010; Hosseinnejad and Jafari, 2016).

In this study, we focused on the antimicrobial and anti-adhesive properties of chitosan, alone or in combination with other compounds (antimicrobial agents, enzymes, antimicrobial peptides, metals, ceramics, and polymers), for the development of surface coatings to prevent the occurrence of IAIs. In recent years, there has been a significant increase in the number of studies concerning CS-based coatings to protect implantable medical devices. However, the currently available data regarding the potential application of CS-based surfaces to prevent biofilm formation on implantable medical devices (IMDs) need to be critically discussed to assist researchers in designing improved CS coatings.

## Results and discussion

### Study selection and characterization

**Figure 3:**
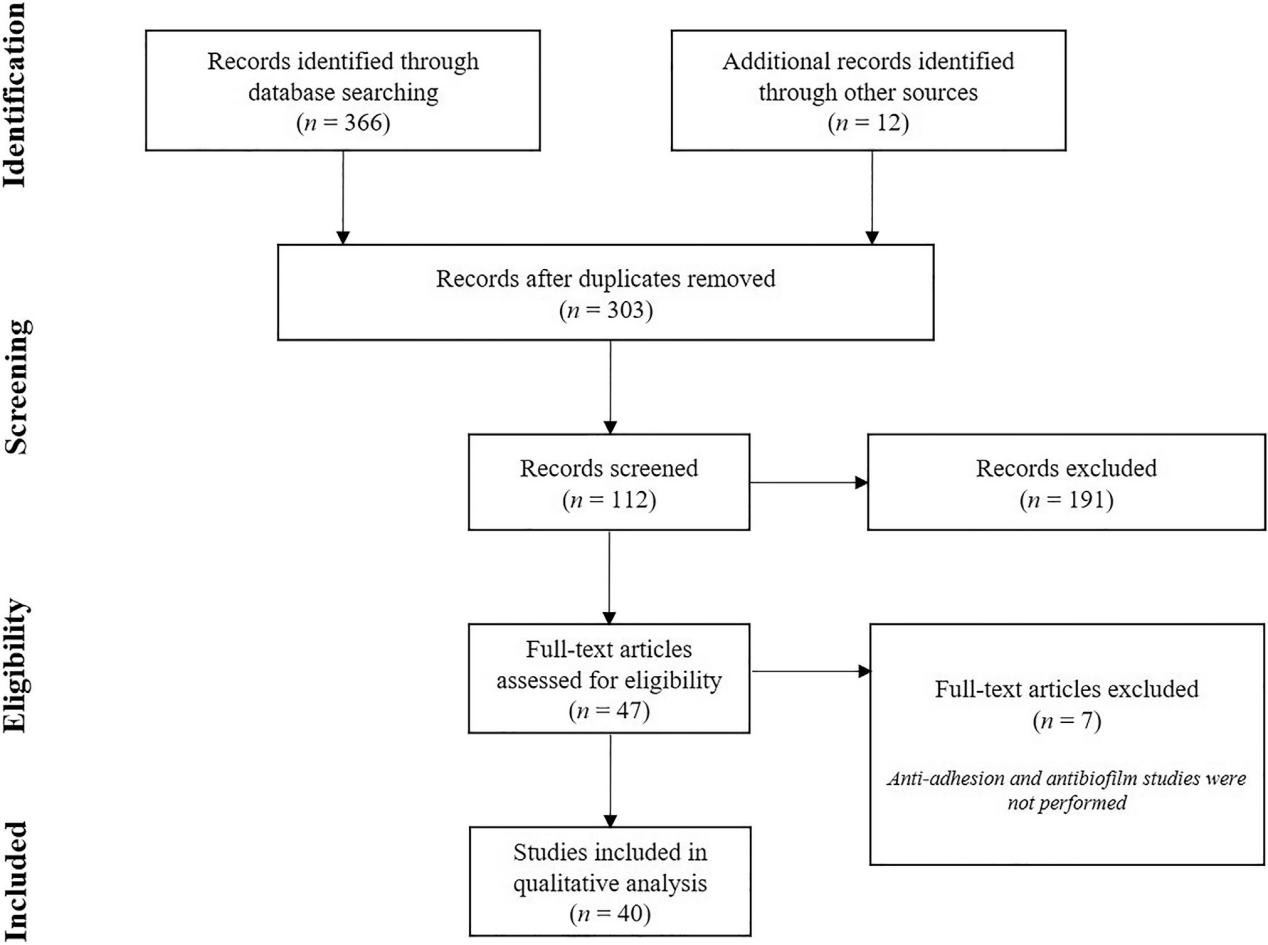
PRISMA flow chart Summary of the literature search based on the PRISMA flow chart (Moher et al., 2009).

A total of 366 studies were found using PRISMA (Preferred Reporting Items for Systematic reviews and Meta-Analysis) search methodology. After the removal of duplicates, 291 studies were considered for screening. Subsequently, 12 studies found by reference list searching were included and a total of 303 studies were screened. During the screening of titles and abstracts, 191 studies were excluded for not fulfilling the inclusion criteria and the remaining 47 studies were selected for a full revision. Among these, 7 studies were excluded because they did not evaluate the performance of CS-based coatings on microbial adhesion and biofilm formation. Therefore, 40 studies were included in the qualitative synthesis (Figure 3).

The use of implantable medical devices is a common and crucial medical procedure applied for both diagnosis and therapeutic purposes. However, the implantation of medical devices is frequently associated with the occurrence of difficult-to-treat infections (Gupta et al., 2016). The incidence of IAIs may be reduced through the optimization of material surfaces. For this reason, in the last two decades, surface modification to provide antimicrobial properties to the materials and devices has been intensively researched (Kara et al., 2014).

Despite the efforts to develop effective antimicrobial coatings, the majority of them may be toxic, trigger microbial resistance during prolonged use, and their clinical application is yet debatable (Carvalho et al., 2020; Gomes et al., 2020; Teixeira-Santos et al., 2021).

Because of their outstanding properties, chitosans have been introduced in the biomedical field to produce improved antimicrobial coatings for IMDs. In this systematic review, 40 studies evaluated the use of CS to prevent microbial adhesion and/or biofilm formation on IMD surfaces. Among the selected articles, 12 studies addressed the antimicrobial and anti-adhesive properties of CS incorporated in different surface materials, and 8 studies focused on the performance of functionalized CS-based coatings. Between the studies addressing the association of CS with other compounds, 10 studies evaluated the conjugation of CS with compounds displaying antimicrobial activity, 5 studies investigated the efficacy of CS in association with different metals or ceramics, and 5 studies evaluated the efficacy of CS associated with polymers.

**Figure 4:**
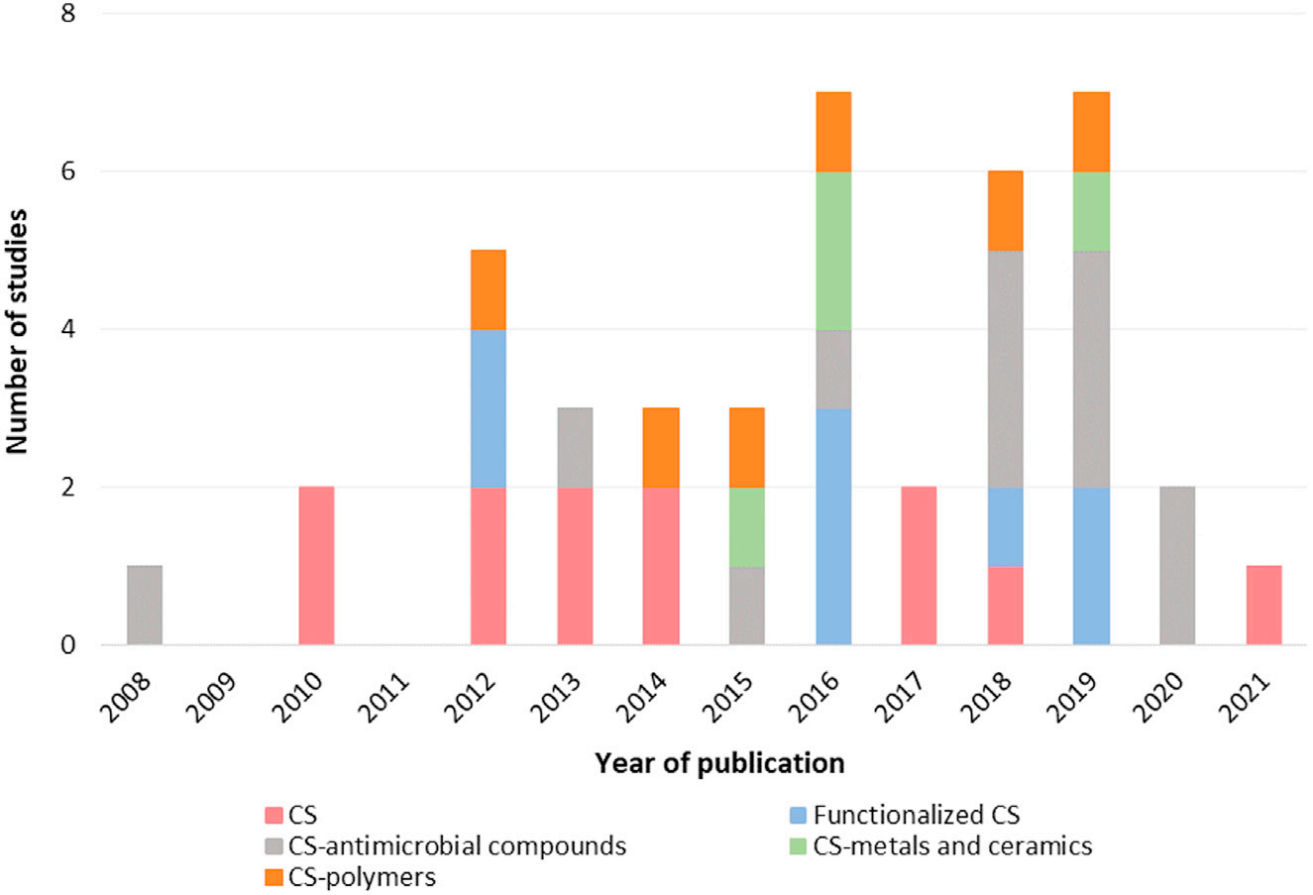
Yearly published studies on chitosan coatings for medical devices Number of published studies addressing the antimicrobial and anti-adhesive properties of chitosan coatings with application in implantable medical devices, by year.

Figure 4 summarizes the research progress on antimicrobial and anti-adhesive coatings based on CS alone or in association with other compounds with application in implantable medical devices. The increasing number of studies in the last five years is considerable, especially for CS associated with antimicrobial compounds and functionalized CS.

Selected studies indicated that chitosan antimicrobial coatings were developed for application on a wide range of IMDs, including central venous catheters (17.5%), orthopedic implants (15.0%), and urinary catheters (12.5%). Moreover, most of the reviewed studies evaluated synthesized coatings against bacteria (82.5%) and only 10% of the studies were performed *in vivo*. This result suggests that a great part of antimicrobial and anti-adhesive CS coatings is yet far from a clinical application. On the other hand, about 30.0% of *in vitro* studies were performed under dynamic conditions, which may increase their predictive value (Ramstedt et al., 2019).

When developing a new coating for biomedical applications, several features, including biocompatibility, hydrophilicity/hydrophobicity, antimicrobial activity, and anti-adhesive properties, should be considered (Francolini et al., 2017). In this review, special attention was given to CS-based coatings with application in IMDs and their antimicrobial and anti-adhesive potential.

#### Non-functionalized chitosan-based coatings

Both *in vitro* and *in vivo* studies have demonstrated the potential of surfaces coated with CS to resist biofilm formation by bacteria and fungi. Table 1 summarizes the performance of CS antimicrobial coatings to inhibit microbial adhesion and biofilm formation on the surface of IMDs.

**Table 1:** Studies demonstrating the efficacy of antimicrobial coatings based on non-functionalized and functionalized chitosans

Coating	Material	Medical application	Species	Major conclusions	Reference
Chitosan	Polyethylene catheters^a^^,^^b^	Central venous catheters and other medical devices	*C. albicans*^f^ *C. parapsilosis*^f^	*In vivo*studies: The fungal burden on CS-treated catheters reduced by 2.5-fold compared with untreated catheters. SEM images demonstrated that biofilms formed on untreated catheters were more extensive than on CS catheters. *In vitro*studies: The viability of *Candida* sp. biofilms reduced 95% after 30 min.	(Martinez et al., 2010b) d
	Polystyrene microtiter plates^b^	Indwelling medical devices	*C. neoformans*^g^	*C. neoformans* biofilms formed in the presence of 0.312 mg/mL CS reduced their metabolic activity by 80%. Mature biofilms also significantly decreased their metabolic activity and viability (*p*< 0.001) after 30 min.	(Martinez et al., 2010a) e
	Silicone^b^	Implantable devices or biosensors	*S. aureus*^h^	Results demonstrated that *S. aureus* biofilm formation was suppressed on CS-coated silicone films. In addition, biofilm metabolic activity on CS films reduced 60% compared to the uncoated films.	(Bulwan et al., 2012) d
	ND^b^	Indwelling medical devices	*K. pneumoniae*^i^	Chitosan (0.0625 mg/mL) induced a biofilm reduction of 86.5%.	(Magesh et al., 2013) e
	Glass^b^	Indwelling medical devices	*C. albicans*^f^	Results demonstrated that 0.0313% CS killed more than 50% of cells in the early and intermediate phases of biofilm development.	(Pu et al., 2014) e
	Polyurethane films^b^	Indwelling medical devices	*P. aeruginosa*^j^ *S. aureus*^h^	Chitosan (0.5%)-coated films reduced *P. aeruginosa* and *S. aureus* biofilm viability to 24.0% and 51.7%, respectively.	(Kara et al., 2014) d
	Polystyrene films^b^	Indwelling medical devices	*A. baumannii*^k^	*A. baumannii* adhered to CS-coated films showed a reduction of biomass and metabolic activity of 93% and 43%, respectively; biofilm biomass and metabolic activity were also inhibited by 80% and 39%, respectively.	(Costa et al., 2017) e
	Foley urinary catheter segments^b^	Urinary catheters	*E. coli*^l^ *K. pneumoniae*^i^	High and low molecular weight CS reduced bacterial adhesion by 70% and 58%, respectively. A low percentage of viable *K. pneumoniae* and *E. coli* cells (about 20%) were recovered from catheters after CS treatments.	(Campana et al., 2017) e
	Foley urinary catheter segments^b^	Urinary catheters	*E. coli*^l^ *K. pneumoniae*^i^	After 48 h, CS-coated catheters were able to reduce *K. pneumoniae* and *E. coli* biofilm formation up to 6.3 Log.	(Campana et al., 2018) e
	Silicone catheters segments^b^	Urinary catheters	*C. albicans*^f^ *S. epidermidis*^h^	CS-coated catheters inhibited *C. albicans*, *S. Epidermis*, and mixed biofilms by 68%, 65%, and 60%, depending on their development stage.	(Rubini et al., 2021) d
Low molecular weight CS	Polyurethane-like catheters segments^b^	Central venous catheters	*A. baumannii*^k^ *C. albicans*^f^ *S. epidermidis*^h^ *S. aureus*^h^	Chitosan (78 mg/mL) reduced the biofilm metabolic activity of *S. epidermidis* by 80.5%, CS (5.0×10^3^ mg/mL) reduced the *C. albicans* biofilm metabolic activity by 87.5% and 90.0% after 24 and 48 h, respectively.	(Cobrado et al., 2012) e
	Polyurethane catheter segments^a^	Central venous catheters	*A. baumannii*^k^ *C. albicans*^f^ *S. epidermidis*^h^ *S. aureus*^h^	The metabolic activity and total biomass of *S. epidermidis* biofilms decreased by 57.6% and 41.3%, respectively, in CS catheters (80 mg/mL CS). In turn, *C. albicans* biofilms reduced their metabolic activity and biomass by 43.5% and 23.2%, respectively, in treated catheters (2.5x10^3^ mg/mL).	(Cobrado et al., 2013) d

**Functionalized-CS**					

Carboxymethyl chitosan	Silicone pre-treated with polydopamine^b^^,^^c^	Medical devices	*E. coli*^l^ *P. mirabilis*^m^	Carboxymethyl CS coating reduced *E. coli* and *P. mirabilis* adhesion by more than 90% after 4 h. Biofilm formation was also inhibited on CS coating under static and flow conditions.	(Wang et al., 2012b) d
	Silicone films^b^	Indwelling medical devices	*C. tropicalis*^f^ *C. parapsilosis*^f^ *C. krusei*^f^ *C. glabrata*^f^	*Candida* spp. biofilm formation was inhibited by 70% for single-species and 73.4% for multi-species biofilms.	(Tan et al., 2016c) e
	Medical-grade silicone^b^^,^^c^	Voice prosthesis	*C. albicans*^f^ *C. tropicalis*^f^ *L. gasseri*^n^ *R. dentocariosa*^o^ *S. epidermidis*^h^ *S. salivarius*^p^	Carboxymethyl chitosan-coated films displayed a surface coverage of 4% less than untreated films.	(Tan et al., 2016b) e
	Medical-grade silicone^b^	Voice prosthesis	*C. albicans*^f^ *C. tropicalis*^f^ *L. gasseri*^n^ *R. dentocariosa*^o^ *S. epidermidis*^h^ *S. salivarius*^p^	Carboxymethyl CS inhibited the adhesion of fungi and bacteria with an efficiency greater than 90%. CS coatings inhibited mixed biofilm formation by 73% and decreased their metabolic activity by more than 60%.	(Tan et al., 2016a) e
	Medical-grade silicone^b^^,^^c^	Indwelling medical devices	*C. tropicalis*^f^ *S. epidermidis*^h^	After 90 min, more than 90% of cells were unable to adhere to carboxymethyl CS (2.5 mg/mL)-coated surfaces. Coated silicone films also inhibited *S. epidermidis*, *C.tropicalis*, and mixed biofilm formation by 64.1%, 66.6%, and 54.7% respectively.	(Tan et al., 2018) e
Quaternised chitosan derivative	Polymethylmethacrylate (PMMA)-based cement^b^^,^^c^	Orthopedic implants	MRSA^h^ *S. epidermidis*^h^	The viability of biofilms formed on functionalized-CS-PMMA surfaces was significantly lower than on PMMA surfaces (*p*< 0.01).	(Tan et al., 2012)^n.d.^
Fatty acid derivatives	Poly(ethylene terephthalate) and butylene dilinoleate (50:50) polymer^b^	Catheters	*E. coli*^l^	*E. coli* colonization on CS/fatty acid derivates coatings was reduced by more than 80%.	(Niemczyk et al., 2019) d
Cathechol	Polyurethane films^b^^,^^c^	Urethral catheter	*E. coli*^l^	During the initial adhesion, the number of live *E. coli* cells significantly decreased in the CS-catechol hydrogel-coated films compared to the bare substrate (85.23% vs. 48.32%).	(Yang et al., 2019) d

MRSA, methicillin-resistant *Staphylococcus aureus.*

n.d., not described.

a *in vivo* study.

b *in vitro* study.

c study performed under hydrodynamic conditions.

d dip coating.

e non-immobilized CS.

f *Candida* sp.

g *Cryptococcus* sp.

h *Staphylococcus* sp.

i *Klebsiella* sp.

j *Pseudomonas* sp.

k *Acinetobacter* sp.

l *Escherichia* sp.

m *Proteus* sp.

n *Lactobacillus* sp.

o *Rothia* sp.

p *Streptococcus* sp.

In 2010, Martinez et al. (2010b) demonstrated the antifungal and antibiofilm efficacy of CS against *Candida albicans* and *C. parapsilosis* biofilms using a central venous catheter model. *In vitro* studies indicated that the viability of *Candida* spp. biofilms reduced 95% after 30 min of exposure, while *in vivo* studies demonstrated that the fungal burden on CS-coated catheters reduced 2.5-fold compared with the untreated catheter. In addition, scanning electron microscopy (SEM) images revealed that biofilms formed on untreated surfaces were more extensive than on CS-coated catheters. The same authors also investigated the effect of CS on the inhibition of *in vitro* biofilm formation by *Cryptococcus neoformans* (Martinez et al., 2010a). Results showed that the addition of exogenous CS to fungal biofilms significantly reduced yeast viability and metabolic activity and prevented cell adhesion on polystyrene surfaces after 30 min of exposure, which is consistent with CS fungicidal activity (Rabea et al., 2003). In 2014, Pu et al. (2014) also found that CS was able to reduce the metabolic activity and the viability of *C. albicans* biofilms in the early and intermediate phases of its development. This effect is the consequence of the physical stress imposed by CS on the fungal biofilm structures due to the permeabilization of cellular membranes, which allows penetration of CS molecules and its binding to DNA with subsequent inhibition of mRNA synthesis (Sudarshan et al., 1992). It is also likely that the interaction between positively charged CS molecules and negatively charged microbial cell membranes leads to the leakage of intracellular constituents, causing cell death (Jung et al., 1999). Moreover, a net positive charge on the fungal surfaces may retain yeast cells in suspension, preventing biofilm formation (Savard et al., 2002). Therefore, these findings suggest that CS is a promising alternative to current antifungal agents and can be used for designing antimicrobial coatings to protect medical device surfaces from fungal biofilm formation (Martinez et al., 2010a, 2010b; Pu et al., 2014).

Likewise, the antimicrobial properties of CS-based coatings have also been demonstrated against a broad spectrum of gram-positive and gram-negative bacteria. A study developed by Bulwan et al. (2012) revealed that CS-coated silicone films were effective in suppressing *Staphylococcus aureus* biofilm formation and reducing the metabolic activity of adhered cells by 60% compared with uncoated films. The surface physical characterization indicated that the immobilization of CS molecules decreased the hydrophobicity and roughness of the synthesized films, making bacterial adhesion unfavourable (Bulwan et al., 2012). Similarly, CS-coated polyurethane (PU) films reduced the number of *Pseudomonas aeruginosa* and *S. aureus* adhered cells by 2–5 Log (Kara et al., 2014). The authors concluded that CS immobilization improved surface hydrophilicity by increasing the number of polar groups, and thus, the bacterial adhesion decreased. In addition, these films reduced *P. aeruginosa* and *S. aureus* biofilm viability to 24.0% and 51.7%, respectively. As with fungal cells, the antibacterial action of CS results from the interaction between positively charged CS molecules and negatively charged cell membranes, which may prevent mass transfer across the cell wall and may lead to leakage of intracellular content (Kong et al., 2008) (Figure 2). Results also suggested that the antibacterial activity of CS-coated PU films was higher for *P. aeruginosa* than *S. aureus*, which may be explained by bacterial surface polarity. The outer membrane of gram-negative bacteria is essentially composed of lipopolysaccharides containing phosphate and pyrophosphate groups which increase the negative charge density on the bacterial surfaces, leading to higher attraction to the positive CS surfaces compared to *S. aureus* cells (Kara et al., 2014). Since then, the potential of CS surfaces to inhibit biofilm formation by gram-negative bacteria has been vastly explored. In 2017, Costa et al. (2017) also demonstrated that polystyrene films coated with CS reduced the biomass and metabolic activity of adhered *Acinetobacter baumannii* cells and biofilms by 93% and 43%, and 80% and 39%, respectively. Campana et al. (2017) showed that segments of Foley urinary catheters coated with either high or low molecular weight (MW) CS were able to reduce *in vitro* bacterial adhesion by 70% and 58%, respectively, and only 20% of viable *Klebsiella pneumoniae* and *E. coli* cells were recovered from CS-treated catheters. In 2018, the same research group evaluated the potential of CS-coated urinary catheters to inhibit bacterial biofilm formation. Results revealed that coated catheters reduced *K. pneumoniae* and *E. coli* biofilm formation up to 6.3 Log (Campana et al., 2018). The potential of CS molecules to inhibit *K. pneumoniae* biofilm formation (86.5% reduction) had previously been demonstrated (Magesh et al., 2013).

Recently, Rubini et al. (2021) studied the performance of CS-coated silicone catheters to inhibit *C. albicans*, *Staphylococcus epidermidis*, and mixed biofilms. Data demonstrated that CS-coated urinary catheters inhibited biofilm formation by 60%–68%, depending on the biofilm development stage (Rubini et al., 2021).

Although both low and high molecular weights (MWs) chitosans have displayed high efficacy to reduce microbial adhesion and biofilm formation on the surfaces of IMDs, some authors focused their attention on the study of low-MW CS. Firstly, Cobrado et al. (2012) showed that PU catheter segments coated with low-MW CS decreased the metabolic activity of *S. epidermidis* and *C. albicans* biofilms by 80.5% and 87.5%, respectively, after 24 h. Given these promising results, the authors also tested the efficacy of CS-coated PU catheters to inhibit *in vivo* biofilm formation (Cobrado et al., 2013). The metabolic activity and biomass of *S. epidermidis* biofilms decreased by 57.6% and 41.3%, respectively, while *C. albicans* biofilms reduced their metabolic activity and biomass by 43.5% and 23.2%, respectively (Cobrado et al., 2013). Comparing both studies, results indicated that the performance of CS-coated catheters decreased when tested *in vivo*, revealing the importance of testing antimicrobial coatings under conditions that mimic the clinic scenarios (either *in vivo* tests or experimental conditions closer to reality, e.g., controlled hydrodynamic conditions, temperature, and culture medium).

In general, CS-based surfaces were able to decrease the fungal biofilm formation up to 65% and reduced the viability of biofilm cells by 95%, reduce the adhesion and biofilm formation of gram-negative bacteria by 87% and 70%, respectively, and decrease the viability and metabolic activity of gram-positive biofilms by 50% and 80%, respectively.

These data show the potential of chitosans to reduce not only the adhesion of human pathogens on medical devices, including urinary and central venous catheters, but also to prevent their re-growth ability.

#### Functionalized chitosan-based coatings

The functionalization of CS molecules has been explored aiming to improve their antimicrobial properties. Table 1 describes different CS functionalizations and their effect on the antimicrobial activity of CS-based coatings.

**Figure 5:**
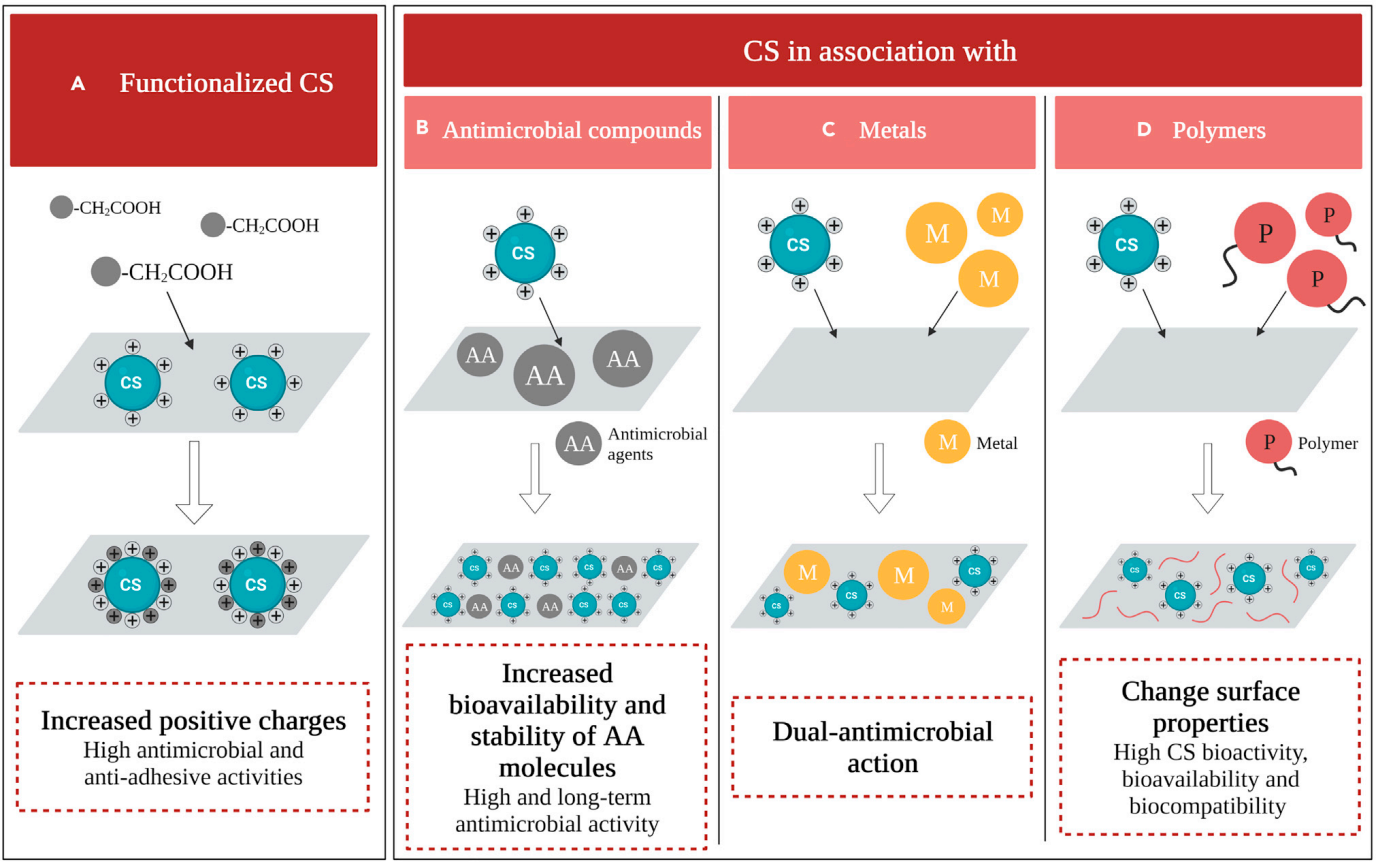
Antimicrobial activity of modified chitosan Antimicrobial effect of coatings based on (A) functionalized CS and CS in association of (B) antimicrobial compounds, (C) metals, and (D) polymers.

Several authors believe that the functionalization of CS molecules by the substitution of the -OH groups with -CH_2_COOH, generating carboxymethyl chitosans (CM-CS) may enhance their intrinsic antimicrobial properties (Figure 5A). In 2012, Wang et al. (2012b) produced silicone films pre-treated with polydopamine and impregnated with CM-CS and evaluated their efficacy to inhibit *E. coli* and *Proteus mirabilis* adhesion and biofilm formation. These films reduced bacterial adhesion by more than 90% after 4 h and were effective in inhibiting biofilm formation under both static and flow conditions (Wang et al., 2012b).

As implant-associated infections are frequently caused by *Candida* spp., Tan et al. (2016c) evaluated the performance of silicone films coated with CM-CS against biofilms formed by single- and multi-*Candida* species. Although some studies have shown that mixed biofilms exhibit enhanced resistance compared with single-species biofilms, these authors indicated that both single- and multi-species biofilms were inhibited by 70%. According to them, CM-CS may interrupt the interactions between different *Candida* species, inhibiting mixed biofilm formation (Tan et al., 2016c). Additionally, the CM-CS-coated silicone films were able to inhibit biofilm formation by multi-fungal and multi-bacterial species (Tan et al., 2016a, 2016b, 2018). After 90 min, CM-CS films inhibited *S. epidermidis* and *C. albicans* adhesion by more than 90% (Tan et al., 2018). Moreover, CM-CS films not only efficiently inhibited fungi and bacteria adhesion (> 90%) but also decreased mixed biofilm formation (70%) and its metabolic activity (60%) (Tan et al., 2016a). These effects may result from the intra- and intermolecular interactions between -COOH and -NH_2_ groups, which lead to an increase in the number of -NH_3_^+^ groups (Fei Liu et al., 2001). Thus, the increased positive charges in CS molecules may potentiate their interaction with negatively charged cell membranes, improving their antimicrobial activity (Figure 5A).

In addition, the functionalization of CS with hydroxypropyltrimethyl ammonium chloride (HACC) increased its water solubility, antimicrobial activity, and biocompatibility, depending on the degree of substitution of the quaternary ammonium (Peng et al., 2010). The effect of polymethylmethacrylate (PMMA) incorporated with HACC was evaluated on the inhibition of methicillin-resistant *Staphylococcus aureus* (MRSA) and *S. epidermidis* biofilms. Data indicated that coated PMMA films significantly reduced biofilm viability through the inhibition of *icaAD* transcription that results in the downregulation of the production of polysaccharide intercellular adhesins (Tan et al., 2012).

A recent study conducted by Niemczyk et al. (2019) also demonstrated that CS-fatty acid derivates (an amphiphilic chitosan derivate) films reduced *E. coli* colonization by more than 80%. Based on previous results, the authors believe that CS-fatty acid derivates are non-nutritive and can inhibit bacterial migration and growth (Niemczyk et al., 2019).

Lastly, Yang et al. (2019) developed catechol-modified CS coatings to inhibit bacterial adhesion on polyurethane films. During initial adhesion, the number of live *E. coli* cells decreased by 37% in catechol-CS-coated films compared to bare polyurethane (Yang et al., 2019). Under physiological or alkaline conditions, catechol groups transform into reactive o-quinones which interact with many other functional groups acting as a natural antibiotic (Yang et al., 2019).

Overall, these results suggest that the modifications introduced on the surface of CS molecules change their physicochemical properties, increasing the potential of CS coatings to reduce microbial adhesion and biofilm formation (by 90% and 70%, respectively).

#### Coatings based on chitosans associated with antimicrobial compounds

As some authors have reported that CS had no antimicrobial activity at pH 7.0 owing to the deprotonation of amino groups and its poor solubility in water (Fei Liu et al., 2001), extensive research has been carried out to improve the CS antimicrobial action and extend its use in a broader pH range through the incorporation of antimicrobial agents or peptides into the CS materials (Yuan et al., 2013). Table 2 lists the studies reporting the efficacy of CS and antimicrobial compound conjugates.

**Table 2:** Studies describing the efficacy of antimicrobial coatings based on chitosans associated with compounds displaying antimicrobial activity

CS-based coatings	Material	Medical application	Species	Major conclusions	Reference
**Antimicrobial agents**					

Rifampicin	CS films^b^^,^^c^	Long-term medical devices	*S. aureus*^f^ *S. epidermidis*^f^	Bacterial cells were not able to grow on CS-rifampicin surfaces after 72 h incubation.	(Cao and Sun, 2009) d
pH-responsive tobramycin-embedded micelles	Polydopamine-modified titanium surfaces^b^	Orthopedic implants	*E. coli*^g^ *S. aureus*^f^	Adhered bacteria were significantly lower (*p*< 0.05) on CS-tobramycin-coated surfaces than on the control group at 4 (1.18%), 12 (0.16%), 24 (0.25%), and 48 h (0.23%).	(Zhou et al., 2018) e
Amoxicillin/clavulanic acid (CoAM)	Silicone^b^^,^^c^	Tympanostomy tubes	*S. aureus*^f^	CS-CoAM-coated silicone films exhibited a high efficacy (> 93%) in the prevention of biofilm formation on the tube surface.	(Ajdnik et al., 2019)

**Disinfectant agents**					

Hyaloronic acid (HA)/triclosan	Modified titanium surfaces^b^^,^^c^	Medical implants	*S. aureus*^f^	Bacteria adhered to CS-HA surfaces lost their viability by 72%, while bacteria attached to the CS-HA/triclosan-coated surface showed a total loss in viability.	(Valverde et al., 2019) d

**Enzymes**					

Lysozyme	Stainless steel surfaces^b^	Medical implants and devices	*S. aureus*^f^	*S. aureus* viability decreased by more than 70% after 2h incubation with CS-lysozyme coatings, and > 95% after 4 h.	(Yuan et al., 2013) d
Proteases	ND^b^	Indwelling medical devices	*L. monocytogenes*^h^ *P. aeruginosa*^i^ *S. aureus*^f^	The antibiofilm activity of proteases was observed after 24 h of incubation; bead mobility was increased with Protease B (36%), Alcalase (57%), and Neutrase (84%).	(Elchinger et al., 2015) e
Cellobiose dehydrogenase (CDH) and deoxyribonuclease I (DNase)	Polystyrene microtiter plates^b^	Indwelling medical devices	*C. albicans*^j^ *S. aureus*^f^	Biofilms of *S. aureus*, *C.albicans*, or mixed species were inhibited by CS nanoparticles-DNase-CDH by 99%, 89%, and 91%, respectively. In addition, these composites caused 80% biofilm disruption on mono- and polymicrobial biofilms.	(Tan et al., 2020)^n.d.^

**Antimicrobial peptides (AMP)**					

Hyaluronic acid (HA)/β-peptide (coumarin-linker-(ACHC-B3hVal-B3hLys)3	Polyethylene catheters^a^^,^^b^	Central venous catheters	*C. albicans*^j^	*In vitro*studies: Biofilms formed on CS-HA-coated catheters reduced their metabolic activity by 80% compared to control. Catheter loading with β-peptide resulted in substantial reductions in biofilm growth (≈10%). *In vivo*studies: Biofilms formed on CS-HA-coated catheters were less robust than those observed on bare catheters. Tubes coated with β-peptide-loaded CS-HA films exhibited either no or very few yeast cells.	(Raman et al., 2016) e
	Titanium surfaces^b^	Orthopedic implants	*S. aureus*^f^	Results demonstrated that coatings loaded with β-peptide prevented the formation of *S. aureus* biofilms for up to 24 days. After 36 days, biofilm viability reduced 60% compared to bare titanium.	(López et al., 2019)
α-helical AMP MSI-78(4-20) (KFLKKAKKFGKAFVKIL)	Gold substrates^b^	Bone implants and other medical devices	*S. epidermidis*^f^	The AMP-chitosan coating did not significantly reduce bacterial adhesion but decreased the viability of adhered cells by 60%.	(Monteiro et al., 2020)

n.d., not described.

a *in vivo* study.

b *in vitro* study.

c study performed under hydrodynamic conditions.

d dip coating.

e layer-by-layer assembly.

f *Staphylococcus* sp.

g *Escherichia* sp.

h *Listeria* sp.

i *Pseudomonas* sp.

j *Candida* sp.

An ideal antimicrobial coating should not only exhibit an initial anti-adhesive ability but also display a long-term prophylactic and antimicrobial performance. In the last years, the association of CS with antimicrobial agents has been explored as an alternative to improve the efficacy of available drugs owing to their synergic mechanisms of action and the chitosans' ability to control the release of active agents (Ajdnik et al., 2019) (Figure 5B).

In this context, Cao and Sun (2009) developed a rifampicin-CS coating for long-term medical devices based on a system that produces localized concentrations of antibiotics on material surfaces using a cationic surfactant. Synthetized films avoided *Staphylococcus* spp. adhesion even after 72 h incubation. This excellent antimicrobial durability is associated with the formation of a stable complex between the CS and anionic antibiotics and its controlled diffusion mechanism (Cao and Sun, 2009). In 2018, Zhou et al. (2018) immobilized tobramycin-loaded micelles embedded in CS/heparin polydopamine on titanium surfaces. The number of adhered bacteria was significantly lower on tobramycin-CS/heparin polydopamine coatings compared to the control (*p* < 0.05). These results indicated that synthesized coatings were capable of long-term tobramycin release in acid environments (pH 4.3), probably because the protonated amino group of CS strengthens electrostatic interaction with micelles keeping them intact and stable (Zhou et al., 2018). Thus, antibiotic-loaded micelles displayed pH-sensitive capability and high-loading efficiency, exhibiting a “long-term release” pattern which is desirable for preventing postoperative infections associated with orthopedic implants insertion (Zhou et al., 2018).

In addition, Ajdnik et al. (2019) coated CS nanoparticles with amoxicillin/clavulanic acid and deposited them onto silicone material. Silicone films coated with amoxicillin/clavulanic acid exhibited a high efficacy (>93%) in the prevention of *S. aureus* biofilm formation up to 1 month of exposure. Therefore, this result indicates the high efficacy of this coating to prevent biofilm formation on tympanostomy tubes (Ajdnik et al., 2019). Lastly, Valverde et al. (2019) synthesized a new coating containing hyaluronic acid–chitosan polyelectrolyte multilayers deposited on modified titanium surfaces for application in medical implants. Subsequently, produced coatings were loaded with triclosan, a disinfectant agent with proven efficacy. Results demonstrated that *S. aureus* cells adhered to hyaluronic acid-CS surfaces lost their viability by 72%, while bacteria attached to the hyaluronic acid-CS/triclosan-coated surfaces completely lost their viability. Multilayers were able to release triclosan within the initial period of bacterial adhesion through the degradation of the outer layers, which are more weakly attached, especially in the first 10 h (Valverde et al., 2019).

Altogether these studies revealed the potential of CS to control the release of active antimicrobial agents on the IMD surfaces with different indwelling times.

Natural defense substances secreted by living organisms have emerged as an attractive class of biocidal agents. Numerous natural enzymes exhibit antimicrobial or antibiofilm abilities with different functions, such as killing microbes by direct contact, interfering with biofilm formation, disrupting the biofilm matrix, inhibiting quorum sensing, or catalyzing reactions to produce antimicrobial compounds (Tan et al., 2020).

In 2013, Yuan et al. (2013) covalently immobilized CS with glutaraldehyde on stainless steel surfaces and conjugated lysozyme on grafted CS to enhance its biocidal effect to inhibit *S. aureus* adhesion and biofilm formation on medical implants and devices. Bacterial viability decreased by more than 70% after 2 h and more than 95% after 4 h of exposure. Results demonstrated that the antibacterial activity of CS may combine with that of the immobilized lysozyme to give rise to a surface with high antibacterial activity, which effectively kills bacteria by contact even under neutral pH conditions (Yuan et al., 2013). It is known that lysozyme damages the cell wall of gram-positive bacteria by catalyzing the hydrolysis of β (1,4) linkages between N-acetyl-muramic acid and N-acetyl-d-glucosamine residues of the peptidoglycan (Vilcacundo et al., 2018). Additionally, several authors have previously reported that lysozyme covalently bound to polymeric films, silicone, and stainless steel exhibited good antimicrobial activity against bacteria (Yuan et al., 2013).

Some biological disrupting enzymes have been used with success against bacterial biofilms. In a previous study, Elchinger et al. (2014) reported that some proteases have activity against *S. aureus* and *S. epidermidis* biofilms. Based on these results, the authors immobilized these enzymes on CS surfaces to obtain films with improved antibiofilm activity (Elchinger et al., 2015). The mobility of *Listeria monocytogenes*, *P. aeruginosa*, and *S. aureus* increased with protease B (36%), alcalase (57%), and neutrase (84%) after 24 h incubation, at pH 7 and 37°C (Elchinger et al., 2015). Results indicated that these enzymes were effective in the early phases of biofilm development, probably owing to their interaction with bacterial adhesins, which are early specific ligands to host factors (Shenkman et al., 2002). Recently, Tan et al. (2020) co-immobilized cellobiose dehydrogenase (CDH) and deoxyribonuclease I (DNase) on CS nanoparticles and tested its antibiofilm activity against *C. albicans* and *S. aureus* monomicrobial and polymicrobial biofilms. CS-DNase-CDH nanoparticles inhibited *S. aureus*, *C. albicans*, and mixed biofilms by 99%, 89%, and 91%, respectively. Additionally, these nanoparticles caused 80% biofilm disruption on mono- and polymicrobial biofilms. These exceptional results were attributed to the triple action of CS, DNase, and CDH. Firstly, CS not only exerts its antimicrobial activity but also improves the stability and usability of co-immobilized enzymes. Secondly, it is known that DNase can disrupt biofilm matrix and enhance its antimicrobial activity when combined with antimicrobial compounds.

Thirdly, CDH generates hydrogen peroxide which possesses the ability to inhibit microbial growth and biofilm formation (Tan et al., 2020).

Another way to improve CS antimicrobial activity consists of the incorporation of antimicrobial peptides (AMPs) into the CS materials. AMPs have been studied as potential new classes of antimicrobial agents. Their mechanism of action involves the disruption of microbial cell membranes, leading to membrane permeabilization, cell lysis, and subsequent cell death (Batoni et al., 2011). Moreover, owing to the lack of a single target, the development of microbial resistance to AMPs and their mimetics may be lower than for traditional antimicrobial agents (Huang and Charron, 2017).

In 2016, Raman et al. (2016) designed a polyelectrolyte multilayer coating (CS and hyaluronic acid) that had inherent antifungal properties as a platform for the immobilization and surface-mediated release of antimicrobial peptides (β-peptide) and tested its *in vitro* and *in vivo* efficacy to inhibit biofilm formation on central venous catheters. *In vitro* studies demonstrated that hyaluronic acid-CS-coated catheters loaded with β-peptide improved the efficacy of hyaluronic acid-CS catheters by 10%. Likewise, in *in vivo* studies, treated catheters loaded with β-peptide exhibited either none or very few yeast cells. These results suggested that the controlled intraluminal release of β-peptide resulted in a substantial reduction of biofilm growth (Raman et al., 2016).

As β-peptides also demonstrated strong antibacterial activity against gram-positive and gram-negative bacteria, (López et al., 2019) evaluated the coating system described above on titanium surfaces to inhibit *S. aureus* biofilm formation. Results indicated that coatings loading with β-peptide prevented *S. aureus* biofilms formation for up to 24 days. After 36 days, biofilm viability reduced 60% compared to titanium surfaces (López et al., 2019).

Recently, Monteiro et al. (2020) covalently bonded α-helical AMP (KFLKKAKKFGKAFVKIL) to a CS coating and tested its performance to prevent *S. epidermidis* adhesion on bone implants and other medical devices. The AMP-chitosan coating did not significantly reduce bacterial adhesion but decreased the cell viability of adhered cells by 60% (Monteiro et al., 2020).

The immobilization of AMPs on CS surfaces provides good surface availability and homogeneous distribution of the AMP on the surface while reducing enzymatic degradation and thus increasing its long-term stability (Figure 5B).

In addition, heparin and CS were immobilized on titanium surfaces to avoid *E. coli* and *S. aureus* biofilm formation on blood-contact medical devices (Zhang et al., 2018). Results demonstrated that the number of bacterial cells on heparin-CS coatings decreased by 26% (Zhang et al., 2018). Although heparin is an anticoagulant, its activity is comparable to antimicrobial agents, which is why it is discussed in this section.

In general, among described strategies, enzymes and antimicrobial peptides have shown promising results in the development of broad-spectrum antimicrobial surfaces for IMDs.

#### Coatings based on chitosan composites

Several studies have reported the synergic association between CS and metallic or ceramic particles to prevent biofilm-related infections (Figure 5C). Table 3 lists the studies reporting the efficacy of CS conjugated with metals and ceramics.

**Table 3:** Studies describing the efficacy of antimicrobial coatings based on chitosans associated with silver and ceramics.

CS-based coatings	Material	Medical application	Species	Major conclusions	Reference
Silver nanoparticles (AgNPs)/polyvinylpyrrolidone (PVP)	Silicone wafers and polyethylene sheets^a^^,^^b^	Medical devices	*E. coli*^e^ *S. aureus*^f^	Chitosan-AgNPs/PVP composites displayed higher antimicrobial activity than polyethylene films (inhibition zone 4 mm vs. 0 mm, respectively). In addition, these films reduced *S. aureus* and *E. coli* adhesion up to 100%.	(Wang et al., 2012a) c
Catechol/Silver nanoparticles	Polyurethane films^a^^,^^b^	Urethral catheters	*E. coli*^e^ *S. aureus*^f^	During the initial adhesion, live *E. coli* cells significantly decreased in the CS-catechol hydrogel-coated films, and further decreased in the CS-catechol/AgNPs-coated films compared to the bare substrate (bare: 85.23%, CS-catechol hydrogel coating: 48.32%, CS-catechol hydrogel coating with AgNPs: 4.70%).	(Yang et al., 2019) c
Zinc oxide/polyaniline (ZnO/PANI) composite	Glass^a^	Indwelling medical devices	*C. albicans*^g^ *P. aeruginosa*^h^ *S. aureus*^f^	CS-ZnO/PANI coatings inhibited *S. aureus* and *P. aeruginosa* biofilm formation by more than 95%. The antimicrobial activity of CS–ZnO/PANI composite against established biofilms resulted in more than 95% inhibition.	(Pandiselvi and Thambidurai, 2015) d
Iron oxide nanoparticles	Polystyrene microtiter plates^a^	Orthopedic implants	*S. aureus*^f^	CS-coated iron oxide nanoparticles decreased the number of biofilm cells up to 3 Log and its metabolic activity by 50% compared to the control.	(Shi et al., 2016)^n.d.^
Apatite	Titanium surfaces^a^	Orthopedic implants	*E. coli*^e^ *S. aureus*^f^	Apatite-CS films reduced biofilms viability by 1 and 2 Log for *E. coli* and *S. aureus*, respectively, after 48 h of exposure.	(Visan et al., 2016)

a *in vitro* study.

b study performed under hydrodynamic conditions.

c dip coating.

d non-immobilized CS.

e *Escherichia* sp.

f *Staphylococcus* sp.

g *Candida* sp.

h *Pseudomonas* sp.

The broad and strong antibacterial activity of silver is already well known. Wang et al. (2012a) produced silver nanoparticles (AgNPs)-doped CS/polyvinylpyrrolidone (PVP) films to reduce *E. coli* and *S. aureus* adhesion on IMDs. In fact, AgNPs-CS/PVP composites were able to reduce bacterial adhesion up to 100% (Wang et al., 2012a).

Yang et al. (2019) incorporated AgNPs into catechol (a natural antibiotic)-CS coatings by adding silver nitrate solutions using the conjugated catechol groups as reducing agents and evaluated the effectiveness of these coatings to inhibit bacterial adhesion on polyurethane films. During initial adhesion, the number of live *E. coli* cells decreased 44% in catechol-CS/AgNPs-coated films compared with catechol-CS-coated films (Yang et al., 2019), confirming the antibacterial activity of silver nanoparticles.

Similar to silver, the majority of inorganic materials possesses superior durability and selectivity and can produce high levels of reactive oxygen species, enhancing microbial cell damages. Zinc oxide nanoparticles (ZnONPs) display antibacterial and antifungal activities at low concentrations (Sawai and Yoshikawa, 2004). The immobilization of ZnONPs on CS-polyaniline composites inhibited *S. aureus* and *P. aeruginosa* biofilm formation and pre-established biofilms by more than 95% (Pandiselvi and Thambidurai, 2015). Iron oxide nanoparticles have also been studied in recent years, demonstrating good antibacterial activity. Shi et al. (Shi et al., 2016) developed CS-coated iron oxide nanoparticles to prevent biofilm formation by *S. aureus* in orthopedic implants. The produced coatings decreased the number of biofilm cells up to 3 Log and biofilm metabolic activity by 50% (Shi et al., 2016).

In addition, Visan et al. (2016) blended CS to biomimetic apatite films, which were deposited by the combinatorial matrix-assisted pulsed laser evaporation method on titanium discs and investigated their efficacy to prevent *E. coli* and *S. aureus* biofilm formation on bone implants. Apatite-CS surfaces reduced biofilms viability by 1 and 2 Log for *E. coli* and *S. aureus*, respectively, after 48 h of exposure (Visan et al., 2016).

In general, the CS association with metals or ceramics to generate antimicrobial coatings effectively reduced fungal and bacterial adhesion (>95%) on medical surfaces.

It is known that the properties of medical surfaces, including hydrophobicity or hydrophilicity, and the presence of surface charges have a high impact on initial microbial adhesion and proliferation (Noimark et al., 2009). For this reason, several studies have proposed surface modification with CS and polymers conjugates to inhibit or modulate microbial colonization. Table 4 describes the efficacy of antimicrobial coatings based on CS associated with polymers.

**Table 4:** Studies demonstrating the efficacy of antimicrobial coatings based on chitosans associated with polymers.

CS-based coatings	Material	Medical application	Species	Major conclusions	Reference
Low molecular weight chitosan hydrogel	Polystyrene plates Polyurethane catheters^a^^,^^b^	Central venous catheters	*C. albicans*^f^ *C. parapsilosis*^f^ *C. glabrata*^f^ *C. tropicalis*^f^ *C. guilliermondii*^f^	*In vivo*studies: Catheter segments soaked with low MW CS-hydrogel significantly impaired the biofilm metabolic activity of *C. parapsilosis* (95.7% ± 3.3). *In vitro*studies: The highest CS-tested concentration (1 × 10^4^ mg/L) caused the biofilm biomass and metabolic activity reductions of all *Candida* spp. up to 99% compared to non-treated biofilms.	(Silva-Dias et al., 2014) d
Poly(lactic-co-glycolic) acid	n.d.^b^	Medical prosthetic devices	*S. aureus*^g^	Biofilms formed on CS-nanocoated surfaces contain at least 2-fold less viable cells compared to uncoated surfaces.	(Iordache et al., 2015)
Hyaloronic acid (HA)	Polyethylene catheters^a^^,^^b^	Central venous catheters	*C. albicans*^f^	*In vitro*studies: Biofilms formed on CS-HA-coated catheters reduced their metabolic activity (80%) compared to control. *In vivo*studies: Biofilms formed on CS-HA-coated catheters were less robust than those observed on bare catheters.	(Tan et al., 2016c) e
	Modified titanium surfaces^b^^,^^c^	Medical implants	*S. aureus*^g^	Bacteria adhered to CS-HA surface lost their viability by 72%.	(Valverde et al., 2019) d
Alginate (anionic polysaccharide)	Silicone	Central venous catheters	*S. aureus*^g^	CS-based coating fully inhibited bacterial growth.	(Mendoza et al., 2018) d

n.d., not described.

a *in vivo* study.

b *in vitro* study.

c study performed under hydrodynamic conditions.

d dip coating.

e non-immobilized CS.

f *Candida* sp.

g *Staphylococcus* sp.

In 2013, Silva-Dias et al. (2014) investigated the *in vitro* and *in vivo* antibiofilm activity of low molecular weight CS hydrogel to prevent *Candida* spp. biofilm formation on central venous catheters. Catheter segments coated with CS hydrogel significantly reduced the metabolic activity (95.7%) of *C. parapsilosis* biofilms formed *in vivo*. Coatings composed of poly (lactic-co-glycolic) acid (PLGA) and CS were also able to decrease by 2-fold the viability of *S. aureus* biofilms compared to uncoated surfaces (Iordache et al., 2015). In fact, PLGA is a relatively hydrophobic polymer that might be used as a scaffold in association with CS, increasing its bioactivity, bioavailability, and biocompatibility (Iordache et al., 2015). Lastly, the association of hyaluronic acid and CS immobilized on polyethylene catheters or titanium surfaces displayed promising results (Batoni et al., 2011; Raman et al., 2016). *In vitro* and *in vivo* studies demonstrated that catheters coated with hyaluronic acid-CS reduced the metabolic activity of *C. albicans* biofilms by 80%, and the biofilms formed on these surfaces were less robust than those formed on bare catheters (Raman et al., 2016). In addition, *S. aureus* cells adhered to hyaluronic acid-CS surfaces lost their viability by 72% (Valverde et al., 2019). Hyaluronic acid is a natural polysaccharide that plays an important role in early- and long-range interactions between cells and substrates owing to its hydrophilic nature (Valverde et al., 2019).

Other authors produced silicone-based catheters coated with CS and alginate (an anionic polysaccharide) (Mendoza et al., 2018). Data demonstrated that there were no *S. aureus* cells on alginate-CS coating, probably because of its anti-adhesive and antimicrobial activities (Mendoza et al., 2018).

In general, the association of CS with polymers resulted in improved antimicrobial and anti-adhesive coatings with desirable properties (e.g., biocompatibility and durability) for medical implants (Figure 4C).

### Coating production techniques

Several techniques can be used to produce antimicrobial coatings. Among the studies included in this review, 40% of them did not indicate the method used to produce the coatings or simply use CS in solution. When CS was used in a non-immobilized way, microbial adhesion and biofilm formation were reduced by 50%–100%, suggesting that CS and its composites are promising in controlling biofilm formation on medical surfaces (Campana et al., 2017, 2018; Cobrado et al., 2012; Costa et al., 2017; Magesh et al., 2013; Martinez et al., 2010a; Pu et al., 2014; Pandiselvi and Thambidurai, 2015; Tan et al., 2016a, 2016b, 2016c, 2018).

In turn, about 38% of studies produced films by dip coating method, which was effective in reducing both microbial adhesion and biofilm formation (20%–100%) (Bulwan et al., 2012; Cobrado et al., 2013; Cao and Sun, 2009; Kara et al., 2014; Martinez et al., 2010b; Mendoza et al., 2018; Niemczyk et al., 2019; Rubini et al., 2021; Silva-Dias et al., 2014; Valverde et al., 2019; Wang et al., 2012a, 2012b; Yang et al., 2019; Yuan et al., 2013; Zhang et al., 2018). Dip coating is a simple approach to coat a substrate generating uniform and thin films (Scriven, 2011). However, although most coatings produced in this way have high antimicrobial activity, in some cases, their effectiveness was lower than with non-immobilized CS. This result may be explained by the coating properties (CS functionalization or used composites) or by the lack of adherence between CS and surface material (Mendoza et al., 2018).

In about 8% of studies, coatings were produced by the layer-by-layer assembly. These films were able to reduce biofilm formation by 35%–90% (Elchinger et al., 2015; Raman et al., 2016; Zhou et al., 2018), displaying, on average, higher antimicrobial activity than those produced by dip coating. The layer-by-layer technique allows the control of surface coverage, film thickness, and the amount of CS adsorbed by changing surrounding chemical conditions, thus influencing the inherent antimicrobial behavior of films (Raman et al., 2016).

Lastly, 15% of studies employed other methods of coating production (e.g., matrix-assisted pulsed laser evaporation, chemical crosslinking, spin coating, and airbrush spraying). Despite having been used in only two studies, the crosslinking technique produced films that efficiently reduced biofilm viability (60%–100%) owing to the controlled release of composites targeting microbial cells (López et al., 2019).

### CS-based coatings for long-term implantable medical devices

One of the key challenges in the development of antimicrobial coatings for implantable medical devices is to extend their stability and efficacy for longer periods to prevent microbial adhesion and proliferation.

According to the European Directives on medical devices 2017/745, a long-term implantable medical device is normally intended for continuous use for more than 30 days. Based on this concept, only 3 of selected studies evaluated the performance of CS-based coatings to inhibit or control biofilm formation for long-term applications (Ajdnik et al., 2019; Raman et al., 2016; López et al., 2019).

Raman et al. (2016) evaluated the long-term ability of central venous catheters coated with β-peptide-loaded CS-HA films to resist fungal challenges and demonstrated its consistent antifungal activity after 49 and 63 days. Likewise, (López et al., 2019) showed that CS films loaded with β-peptide were able to reduce *S. aureus* biofilm viability by 60% compared to bare titanium even after 36 days of exposure.

Ajdnik et al. (2019) assessed the performance of silicone films coated with CS-amoxicillin/clavulanic nanoparticles for preventing *S. aureus* biofilm formation in tympanostomy tubes and demonstrated its high effectiveness (99.75% antibiofilm activity) up to 1 month of exposure.

Altogether, these results demonstrated the potential of CS to control the release of antimicrobial compounds on the surfaces of long-term IMDs, maintaining their stability, activity, and durability.

### Biocompatibility of CS-based coatings

Implantable medical devices require biocompatible coatings to ensure proper performance and patient safety. Among the studies included in this review, about 38% of them evaluated the cytotoxicity of CS-based coatings against human cells (Iordache et al., 2015; Martinez et al., 2010a, 2010b; Mendoza et al., 2018; Niemczyk et al., 2019; Panwar et al., 2016; López et al., 2019; Rubini et al., 2021; Silva-Dias et al., 2014; Tan et al., 2012, 2020; Wang et al., 2012a, 2012b; Zhang et al., 2018; Zhou et al., 2018). Results indicated that human cells exposed to CS coatings displayed viability percentages in the range of control samples (>95%), suggesting that these coatings are non-toxic, exhibit good biocompatibility, and are suitable for implantable medical devices (Iordache et al., 2015; Martinez et al., 2010a; Mendoza et al., 2018).

## Methods

### Search strategy, inclusion criteria, and data extraction

This systematic review was conducted following the PRISMA statement (Preferred Reporting Items for Systematic reviews and Meta-Analysis) guidelines (Moher et al., 2009). The search was performed until 1 April 2021 in PubMed, Cochrane, Scopus, and Compendex libraries through the combination of the following keywords: “chitosan”, “antimicrobial”, “antibiofilm”, “adhesion”, “biofilm”, and “medical applications”. Peer-reviewed full-text articles in English published since January 2000 concerning the antimicrobial activity of chitosan-based coatings for application in implantable medical devices were assessed for eligibility. The adopted inclusion criteria for qualitative synthesis were as follows: (i) the application of chitosan to prevent microbial adhesion and/or biofilm formation on the surfaces of implantable medical devices; (ii) the antimicrobial and anti-adhesive properties of chitosans in association with other compounds for application in implantable medical devices; and (iii) original articles. The exclusion criteria consisted of articles that did not evaluate the effect of chitosan-based coatings to inhibit microbial adhesion or biofilm formation.

Two reviewers (RTS and ML) independently applied the inclusion and exclusion criteria, and any differences were resolved by consensus.

Information regarding study design, CS coatings, surface materials, medical applications, pathogens, used methodologies, and obtained outcomes was gathered.

## Conclusions

The development of improved chitosan-based antimicrobial coatings for implant surfaces is an attractive strategy to reduce the IAIs incidence and improve their treatment.

Most of the selected studies investigated the efficacy of non-functionalized and functionalized CS molecules and developed antimicrobial coatings with a broad spectrum of activity against gram-negative and gram-positive bacteria and yeasts. Numerous innovative strategies were also used to improve the CS antimicrobial action, extend its use in a broader range of physiological conditions, and increase its durability and biocompatibility. Among the described strategies, the conjugation of CS molecules with compounds displaying antimicrobial activity seems to be the most promising approach because studies revealed the high potential of CS to control the release of active antimicrobial agents or peptides on coated surfaces for long periods. In fact, these coatings promote a substantial reduction of biofilm growth and may be applied for long-term IMDs. The CS-polymer coatings follow in terms of efficacy and desirable properties. Lastly, coatings based on CS conjugated with metals or ceramics were effective in reducing microbial adhesion on medical surfaces, but there is still important information to be unraveled about its biocompatibility and long-term stability.

Although these studies contain promising results, only a small fraction was performed *in vivo*, suggesting that most antimicrobial and anti-adhesive CS coatings may still be far from clinical application. Furthermore, the long-term stability and biocompatibility of the synthesized coatings were assessed in a limited number of studies, indicating that further research is needed in order to apply these coatings in IMDs.
